# Handgrip strength in old and oldest old Swiss adults – a cross-sectional study

**DOI:** 10.1186/s12877-018-0959-0

**Published:** 2018-11-06

**Authors:** Julia Wearing, Peter Konings, Maria Stokes, Eling D. de Bruin

**Affiliations:** 1 Faculty of Health, Medicine and Sciences, School for Public Health and Primary Care, University Maastricht, Minderbroedersberg 4-6, Maastricht, LK 6211 The Netherlands; 2 Adullam Stiftung, Mittlere Strasse 15, 4056 Basel, Switzerland; 3 Geriatrische Klinik St. Gallen, Rorschacher Strasse 94, 9000 St. Gallen, Switzerland; 4 Faculty of Health Sciences, University of Southampton, Building 45, Highfield Campus, Southampton, SO17 1BJ UK; 5 Insitute of Human Movement Sciences and Sport (IBWS) ETH, Department of Health Sciences and Technology, HCP H 25.1, Leopold-Ruzicka-Weg 4, 8093 Zürich, ETH, Zurich, Switzerland; 6 Division of Physiotherapy, Department of Neurobiology, Care Sciences and Society, Karolinska Institutet, Stockholm, Sweden

**Keywords:** Grip strength, Aged, Geriatric assessment

## Abstract

**Background:**

Handgrip strength is indicative of overall physical health and mobility in the elderly. A reduction in strength below a certain threshold severely increases the risk of mobility limitations and is predictive for adverse outcomes such as dependence in daily activities and mortality. An overview of age- and geography- specific handgrip strength values in older adults provide a reference for further investigations and measures in clinical practice to identify people at risk for clinically meaningful weakness. The aim of this study was to evaluate handgrip strength in the Swiss-German population aged 75 and over.

**Methods:**

In a cross-sectional study, maximal isometric handgrip strength of the dominant hand was evaluated in 244 Swiss people aged 75 years and over (62.7% women), with mean age (SD) of 84.5 (5.6) years in men and 83.1 (5.9) years in women. Demographic data and information about comorbidities, medication, fall history, global cognitive function, self-reported physical activity and dependence in activities of daily living were collected, and correlated with grip strength measures. Age- and gender specific grip strength values are reported as means, standard deviations and standard error of mean.

**Results:**

Sex-stratified handgrip strength was significantly lower with advancing age in men (*p* < .01), from 37.7 (6.5) kg to 25.6 (7.6) kg and in women (*p* < .01) from 22.2 (4.0) kg to 16.5 (4.7) kg. Handgrip strength in our sample was significantly higher than in Southern European countries. Handgrip strength was independently associated with age, height and ADL dependence in men and women. Overall, 44% of men and 53% of women had handgrip strength measures that were below the clinically relevant threshold for mobility limitations.

**Conclusion:**

This study reports the age- and sex-stratified reference values for handgrip strength in a representative sample of the Swiss population, aged 75–99 years. Although grip strength decreased with advancing age in both sexes; the relative decline was greater in men than women. Nonetheless men had significantly higher grip strength in all age groups. While the Swiss population sampled had greater grip strength than that reported in other European countries, about 50% were still classified as at risk of mobility limitations.

## Background

Muscle strength is an important determinant of healthy aging [1]. A reduction in muscle mass and strength is known to impair body function and can have substantial consequences directly for the individual but also for economic costs [2]. Impairment in body function initially results in difficulties in performing common daily activities such as carrying household items; however, once body strength drops below a clinically relevant threshold, mobility limitations increase and can affect independence in basic daily life activities [3, 4]. Loss of independence requires the support of care-givers and often leads to social withdrawal and negatively effects on wellbeing and quality of life [2]. Early detection of low muscle strength in the elderly may help identify those at risk of mobility limitations and apply interventions to avoid or slow down the spiral of negative outcomes.

Muscle deterioration in old age is primarily explained by neural and muscular decline due to the aging process and concomitant physical inactivity and malnutrition [1]. However, mobility-limiting muscle weakness can potentially be counteracted or improved through preventive exercise and rehabilitation respectively [1, 5]. Increased physical activity and resistance exercise have been shown to improve muscle strength and -function even in older people with severe disability [6].

An easily applicable measure of muscle strength is a handgrip strength test. Maximal isometric handgrip strength, measured with a dynamometer in a standard procedure, has high to excellent inter-tester and test-retest reliability [7]. Low handgrip strength is indicative for decline of upper extremity strength [8] and lower extremity function [9] with high predictive value of adverse outcomes [10]. In clinical research, grip strength is often used in detection of age-related changes of muscle strength, associated with sarcopenia [11] and frailty [12]. Low grip strength is related to poor mobility of the elderly [13] and dependence in activities of daily living [14], and even predicts decline in body function and mortality [10].

Moreover, measurement of grip strength alone has been proposed to be a reliable marker of frailty [15] and has, in combination with gait speed, a positive predictive value of 87.5% to identify frailty [16]. Handgrip strength in older adults is considered a meaningful measure of current physical decline and future outcome by the World Health Organization [17].

Theoretical models of demographic trends show an increasing average life expectancy in industrial countries [18]. Particularly the percentage of older adults over 65 years will expand, in Switzerland from 18% in 2015 to 26% in 2045, whereas the old and oldest old age group (75 years and over) will increase the most. 60% of the over 80 year olds in Switzerland seek private help or live in old peoples- or nursing- homes because of limitations in basic and/or complex activities in daily living [19, 20].

The severity of consequences of age-associated muscle weakness provides significance to determine strength across all ages, particularly in the 75 years and over group. In this context, “hand-grip dynamometry can be considered a fundamental element of the physical examination of patients, particularly if they are older adults” [21].

Although many studies have collected grip strength data in the elderly, only few have systematically assessed grip strength in the most advanced age groups spanning the range from 90 up to 100 years and over [22, 23]. To the best of our knowledge, so far only one study evaluated grip strength in the Swiss population [24]. Since average grip strength differs depending on geographic regions [25, 26], an extension of Swiss reference values is important for interpreting region-specific handgrip strength measures in clinical practice.

This study aimed to assess handgrip strength in the Swiss population aged 75 years and above to provide reference values for further investigations and measures in clinical practice.

## Methods

### Study design

A cross-sectional study of handgrip strength involving older people living in two different urban regions (Basel and St Gallen) of the German-speaking part of Switzerland was undertaken. Recruitment targeted community-living elderly, as well as those dwelling in assisted living apartments, and residential aged-care/nursing homes to ensure a broad representative sample of the general older population. Participants meeting the following inclusion criteria were eligible for the study: male and female adults aged 75 years or older, able to follow verbal instructions in German, able and willing to sign informed consent. Participants were excluded from the study based on the following criteria: self-reported upper extremity pain, aching or stiffness of the upper extremity on most days (over 50%) of the past month, injury or surgery or acute diseases of upper extremity within the past 6 months, and inability to follow the procedures of the study.

### Sample size

Participant numbers (*n* = 240) were estimated a priori based on previously published grip strength data for Swiss older adults. The number would be sufficient to detect a 30% difference in grip strength for each 10-years age group cluster at an alpha (ɑ) level of .05 and with 80% power (β = .20).

### Data collection and methods

Prior to data collection, research assistants at both study sites were trained in conducting the interview of the participants and in using the study equipment for measuring handgrip strength according to the study protocol. Factors previously shown to influence handgrip strength including demographic characteristics, medication and fall history, osteoarthritis of the hands, global cognitive function, physical activity and dependence in activities of daily living were also collected.

Information about the intake of sedative medication, fall history and osteoarthritis of the hands were self-reported by the participants.

Measurements of body height were made to the nearest centimeter with a stadiometer and body weight was measured to the nearest kilogram on a digital weigh scale.

Global cognitive function was evaluated with the Mini Mental State Exam (MMSE) [27] and expressed as a score out of 30. The MMSE has a reported sensitivity of 77% and specificity of 91% in detecting cognitive impairment in older, community dwelling, hospitalized and institutionalized adults [28].

Independence in daily activities was assed via two questionnaires; the Barthel Index, which assesses basic activities of daily living (ADL) [29], and the Lawton Scale which evaluates instrumental activities of daily living (IADL) such as telephone use, shopping and food preparation [30]. Both self-rated assessments are widely used in elderly cohorts and have been shown to have high levels of reliability (intraclass correlation coefficient and Cronbach’s alpha 0.9) [31, 32]. The questionnaires were conducted as interviews and categorized participants as independent (when all activities were scored highest), dependent in IADL (when at least one complex activity was rated with 0 points) or dependent in ADL (when at least one basic activity was rated with less than maximum score).

Physical activity was assessed with the Freiburg Questionnaire of Physical Activity; a self-reported questionnaire comprised of 8 items evaluating occupational, household, and leisure activities during the previous 7-day/30-day period [33]. Energy cost per week was quantified using a specific coding scheme that classifies physical activity by rate of energy expenditure [34, 35].

Handgrip strength was assessed using a hydraulic hand dynamometer (Jamar®) according to the standardized protocol recommended by the American Society of hand therapists [36]. The participant was seated in a chair without arm support, and with their hips flexed at 90° and feet resting on the floor. The elbow of the test arm was flexed to 90°, the forearm in neutral, and the wrist positioned at 15–30° of extension (dorsiflexion) and 0–15° of ulnar deviation. The examiner supported the base of the dynamometer for testing and the second smallest dynamometer handle position was used. Following a demonstration of the protocol, the participant was asked to squeeze the handle with as much force as possible for three seconds. Three repeated trials were recorded for both hands with a rest period of at least 15 s between trials. The maximum value of the three trials was used for analysis and data presentation. To enable comparison of results with those of other authors, the mean value of three trials was also reported. Hand dominance was self-reported by the participant based on their preferred hand use in activities including writing and brushing teeth, according to the Edinburgh Handedness Inventory [37].

IBM SPSS Statistics, Version 23 was used for statistical analysis. Descriptive statistics for categorical variables were expressed in percentage frequency distribution, for continuous variables mean and standard deviation was used. Grip strength of the dominant hand (mean and maximum value of three trials) was reported as means and standard deviations (SD) and standard error of mean (SEM) for men and women by age group. Measures of handgrip strength controlled for height were also presented. Spearman’s correlation coefficient and multiple regression analysis was used to calculate relationships of grip strength with demographic data and information about comorbidities, medication, fall history, global cognitive function, self-reported physical activity and dependence in activities of daily living. Multivariate analysis of variance with Bonferroni-adjusted post-hoc analysis was used to detect strength differences between age groups and sexes.

This manuscript adheres to reporting guidelines for cross-sectional studies [38].

## Results

A total of 244 participants were recruited in the period from June 2016 to march 2017, including 164 people from the canton Basel and 80 from the canton St Gallen. There was no statistically significant difference in mean age- and sex-stratified grip strength between the two sites (*p* = .24).

Characteristics of participants (62.7% female) and mean grip strength are shown in Table 1. There were no differences between sexes for age, global cognitive function, dependence in activities in daily living, amount of people living in assisted-living facilities, taking sedative medication or experiencing a fall but males were significantly taller, heavier, stronger, more physically active and had less hand osteoarthritis than females.

**Table 1:** Participants characteristics

Characteristic	men mean (SD) or %	women mean (SD) or %
age (years)	83.1 (5.6)	84.5 (5.9)
height (m)	1.73 (0.7)	1.59 (0.7)*
weight (kg)	75.2 (10.4)	63.3 (13.2)*
handgrip strength (kg)	32.0 (8.2)	19.4 (4.3)*
Global cognitive function (points)	27.6 (2.4)	26.9 (3.1)
Physical activity (kcal/week)	1467.4 (1435.9)	828.2 (1005.1)*
ADL dependence		
*in instrumental activities of daily living*	18.2	16.0
*in instrumental and basic activities of daily living*	17.0	23.7
Living in assisted-living facilities/nursing homes	6.8	9.6
Medication	23.9	34.0
Osteoarthritis in hands	11.4	27.6*
Fall history	52.3	64.7

*significant difference between values of men and women with *p* < .05

Handgrip strength in men significantly correlated with age (ρ = −.41, *p* < .01), height (ρ = .31, *p* < .01) and ADL dependence (ρ = −.42, *p* < .01). After multiple regression analysis, all three variables showed independent association with grip strength, with a regression coefficient of −.4 for age in years and .3 for height in m and − 7.5 for ADL dependence. In women, handgrip strength significantly correlated with age (ρ = −.49, *p* < .01), weight (ρ = .20, *p* < .02), height (ρ = .30, *p* < .01) and ADL dependence (ρ = −.49, *p* < .01). After multiple regression analysis, only age, height and ADL dependence were independently associated with grip strength, with a regression coefficient of −.2 for age in years and .1 for height in m and − 2.8 for ADL dependence.

**Fig. 1:**
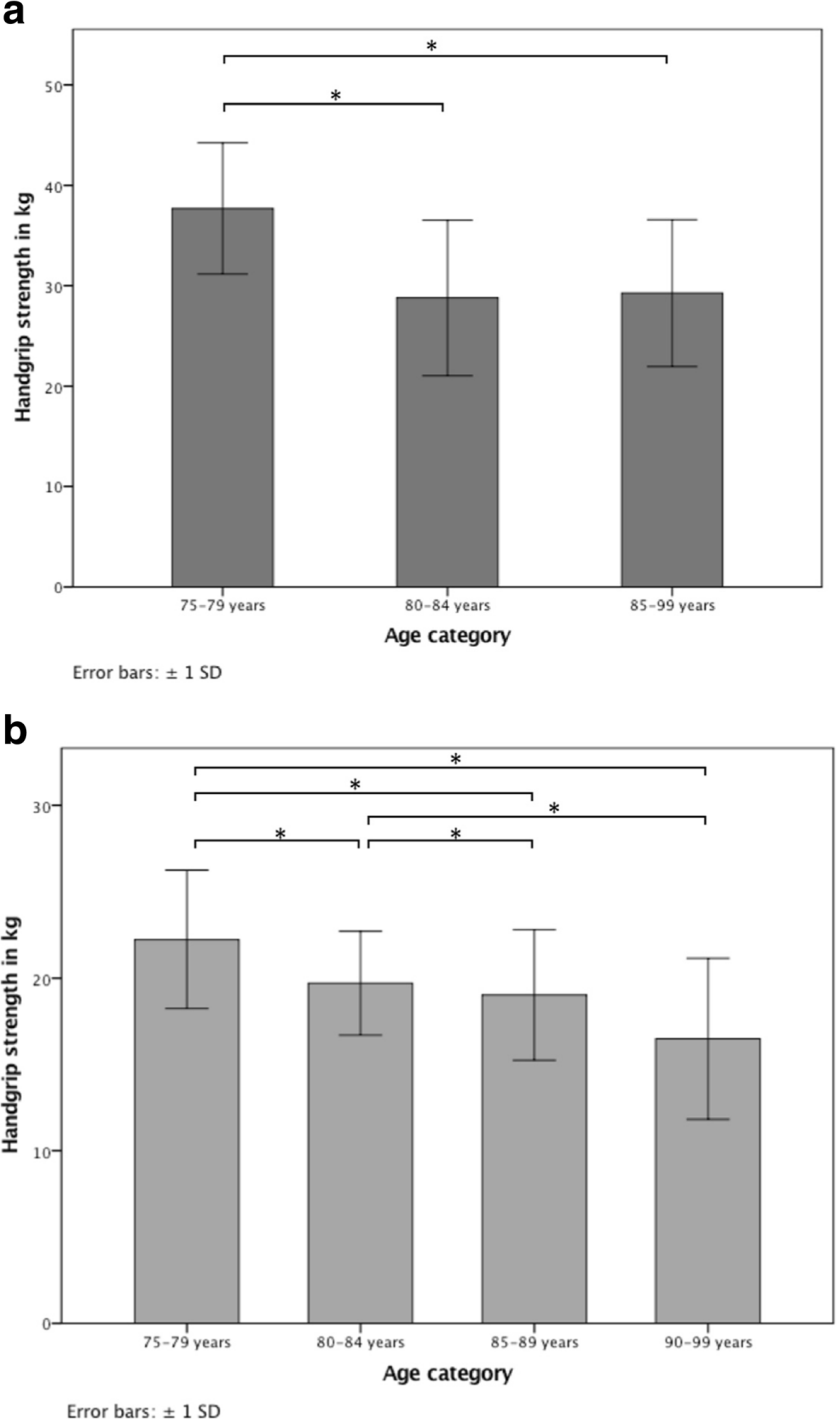
**a** Maximum handgrip strength (kg) in men of the Swiss population 75 years and over. * Significant difference in grip strength (*p* < .05). **b** Maximum handgrip strength (kg) in women of the Swiss population 75 years and over. * Significant difference in grip strength (*p* < .05)

For presentation of handgrip strength results as reference values and to aid comparisons with previous research, mean as well as maximum values of three trials were given for participants categorized into age groups, with each group including at least 20 individuals: age groups 75–79 years, 80–84 years, 85–89 years and 90–99 years for women and age groups 75–79 years, 80–84 years, 85–99 years for men. Age distribution is presented in Tables 2 and 3. Handgrip strength was calculated for men and women separately. Handgrip strength results in kg of male and female participants are presented with and without controlling the values for height (assuming all males and females had the same height within their respective groups) in Tables 4, 5, 6 and 7, respectively. Unadjusted maximum handgrip strength is graphically shown in Fig. 1.

**Table 2:** Number of male participants per age group in absolute (n) and percentage values (%)

Age (years)	75–79	80–84	85–99
Men			
*Absolute n*	30	28	30
*percentage %*	34.1	31.8	34.1

**Table 3:** Number of female participants per age group in absolute (n) and percentage values (%)

Age (years)	75–79	80–84	85–89	90–99
Women				
*absolute n*	37	45	37	37
*percentage %*	23.7	28.8	23.7	23.7

**Table 4:** Height-adjusted handgrip strength (kg) of the dominant hand in men, categorized in age groups

Age (years)	75–79	80–84	85–99
Men			
*mean of 3 trials*	35.9 ± 6.3	27.5 ± 7.7	28.1 ± 7.1
*max of 3 trials*	37.7 ± 6.5	28.8 ± 7.7	29.6 ± 7.2

Handgrip strength is presented as maximum value of three trials and mean value of three trials ± SD

**Table 5:** Height-adjusted handgrip strength (kg) of the dominant hand in women, categorized in age groups

Age (years)	75–79	80–84	85–89	90–99
Women				
*mean of 3 trials*	21.0 ± 3.9	18.2 ± 3.0	18.0 ± 3.7	15.4 ± 4.6
*max of 3 trials*	22.2 ± 4.0	19.7 ± 3.0	19.0 ± 3.8	16.5 ± 4.7

Handgrip strength is presented as maximum value of three trials and mean value of three trials ± SD

**Table 6:** Handgrip strength (kg), unadjusted to height, of the dominant hand in men, categorized in age groups

Age (years)	75–79	80–84	85–99
Men			
*mean of 3 trials*	35.9 ± 6.3 (1.2)	27.5 ± 7.7 (1.5)	27.8 ± 7.3 (1.3)
*max of 3 trials*	37.7 ± 6.5 (1.2)	28.8 ± 7.7 (1.5)	29.3 ± 7.3 (1.3)

Handgrip strength is presented as maximum value of three trials and mean value of three trials ± SD (SEM – standard error of mean)

**Table 7:** Handgrip strength (kg), unadjusted to height, of the dominant hand in women, categorized in age groups

Age (years)	75–79	80–84	85–89	90–99
Women				
*mean of 3 trials*	21.0 ± 3.9 (0.6)	18.2 ± 3.0 (0.4)	18.1 ± 3.7 (0.6)	15.3 ± 4.6 (0.8)
*max of 3 trials*	22.2 ± 4.0 (0.7)	19.7 ± 3.0 (0.4)	19.0 ± 3.8 (0.6)	16.5 ± 4.7 (0.8)

Handgrip strength is presented as maximum value of three trials and mean value of three trials ± SD (SEM – standard error of mean)

Analysis of variance for maximum handgrip strength in men showed that age group 75–79 was significantly stronger than age group 80–84 (*p* < .01, 95% CI 4.3–13.5) and 85–99 (*p* < .01, 95% CI 3.9–13.0). Handgrip strength between age group 80–84 and 85–99 did not differ significantly (*p* = 1.0, 95% CI -5.1 - 4.1).

Difference in mean handgrip strength between the youngest two age groups (75–79 and 80–84) was 8.9 ± 1.8 kg (23.6 ± 4.7%), and 8.4 ± 1.8 kg (22.3 ± 4.7%) between age groups 75–79 and 85–99.

In women, analysis of variance for handgrip strength was significantly higher in age group 75–79 than in the other three groups (80–84, *p* = .02, 95% CI 0.2–4.8, 85–89, *p* < .01, 95% CI 0.8–5.6; and 90–99, *p* < .01, 95% CI 3.4–8.2). Handgrip strength of age group 80–84 and 85–89 were significantly stronger than age group 90–99 (*p* < .01, 95% CI 0.9–5.5; *p* = .03,95% CI 0.1–5.0), but did not differ from each other (*p* = 1.0).

Difference in mean handgrip strength between the youngest two age groups (75–79 and 80–84) was 2.5 kg ± 0.9 (11.3 ± 4.1%), and 5.8 ± 0.9 kg (26.1 ± 4.1%) between the youngest and the oldest (75–79 and 90–99).

For identification of participants with clinically meaningful weakness, handgrip strength was classified in three categories: weak, intermediate and normal, according to cut-off values published by Alley et al. [3]. Allocation of participants to individual categories is presented in Table 8. Men and women show equal percentage distribution for each category except for intermediate strength, where women were significantly higher. About 50% of both sexes have normal strength and 50% were categorised as having reduced strength (category weak and intermediate). The percentage distribution of participants living in assisted-living facilities did not differ between sexes but significantly differed between categories. In men and women, more people categorised as weak lived in assisted-living facilities than people with intermediate or normal strength.

**Table 8:** Classification of participants (%) into three handgrip strength categories

	men (%)	women (%)
Weak (men < 26 kg, women < 16 kg)	22.7	18
*assisted-living*	20ˆ	21ˆ
*community-living*	80	79
Intermediate (men ≥26 < 32 kg, women ≥16 < 20 kg	21.6	35.3*
*assisted-living*	7	10
*community-living*	93	90
Normal (men ≥32 kg, women ≥20 kg)	55.7	46.7
*assisted-living*	4	5
*community-living*	96	95

Three groups for handgrip strength: weak, intermediate and normal. Percentage of people living in assisted-living facilities per group is presented in %

*Significant difference between men and women, *p* < .05

ˆSignificant difference of people living assisted (%) between strength categories, *p* < .05

## Discussion

The present study evaluated handgrip strength in a sample of Swiss individuals aged 75 years and over to provide reference values for further investigations and measures in clinical practice. Our study results showed handgrip strength values confirm and equal previously published data in the Swiss population 75–85 years [24] and confirmed the validity of provided reference values for this geographic region. For the first time, additional reference values for women specifically for the age groups 85–90 and 90–99 years of the Swiss population are presented. There is a need to provide reference values for screening tests, such as handgrip strength, particularly for this age group 85 and over, as with increased life expectancy, the risk of poor health increases. The old and oldest age groups are expected to increase the most; age-specific handgrip strength helps to identify individuals with low strength and to plan specific preventive health services to lower the risk of mobility limitations and dependence in activities of daily living.

Compared to grip strength data of a previously published Swiss sample (up to 85 years of age) [24], the older people in our study presented with comparable strength values (mean of three trials) in all age groups, except from women aged 75–79 years who were significantly weaker in the present study. Where Werle et al. included community-living older adults and elderly living in senior residences, our sample included nursing home dwellers as well. It is possible, therefore, that our sample had a lower level of physical condition than participants in the Werle et al. study. Therefore, handgrip strength could be expected to be lower in our sample as seen in women aged 75–79 years. The age group 85+ reported in the study of Werle et al. could not be compared to our sample since information on average age of their 85+ cohort was not provided.

In comparison to handgrip strength values published by other authors who included a random sample of the general nonagenarian population [22, 23], mean handgrip strength of the 90–99-year-old participants were significantly higher than in Southern France and Italy. These findings are consistent with previous comparisons among different European countries showing a North-South slope [22, 25]. Contrasting our results of the oldest women with studies of two cohorts from Denmark (women mean age 100 years, 92–92 years respectively) who included volunteers of oldest old people registered in the national civil registration system [22, 23], women of our Swiss sample were significantly stronger. The difference could be due to variances in mean age, with women of one Danish cohort being 7 years older on average, as well as due to higher percentage of the participants living in assisted living facilities/nursing homes in both Danish samples (30.6% [22] and 47.6% [23] versus 8.4% in our sample).

The differences in age- and gender-specific grip strength among different countries likely vary due to e.g. birth weight, lifestyle and health care in the elderly [25]. In the Swiss population, these factors are above average on international comparison, which might contribute to the higher grip strength observed in the elderly Swiss. More specifically, the average birth weight (3.3 kg) of Swiss newborns in 2016 [39] corresponds with the average value of international standards for newborns [40]. However, at 83.3 years, the Swiss population had the second highest life expectancy at birth in 2016 [41]. Moreover, 56% of the population aged 75 years and over met the WHO-recommendations for physical activity in 2016, and therefore were within the highest quartile of the prevalence range (20–60%) of physical activity in older adults [42]. Remarkably, Switzerland has the highest social and economic wellbeing of older people worldwide, considering income and health status, education and employment, and enabling environment [43], which may be important preconditions for remaining active in old age.

Another finding of our study, consistent with previous research in the elderly [14, 25], was that handgrip strength in men and women was independently associated with age, height and ADL dependence. Handgrip strength did not correlate with Body Mass Index (BMI), probably owing to homogenous BMI values across sexes and age groups. Since percentage of muscle and fat mass was not specified in the study participants, no conclusion about association between grip strength and body composition could be drawn. Therefore, age-specific grip strength values were demonstrated only with and without adjustment to height and not BMI. Handgrip strength decreased significantly with age in men and women. Between 75 and 99 years, men demonstrated a greater decrease in strength than women but had still higher overall values even in the oldest age group. When considering the entire age range (75–99 years), the largest reduction in grip strength occurred in men in their early 80’s while the biggest difference in women’s strength appeared in their early 90’s. The finding is consistent with a longitudinal study of Danish older adults, in which males lost handgrip strength more rapidly than females but were still stronger in absolute values [44] and less dependent in daily living [23]. As more women of the oldest age group in the current study were dependent in daily activities than men (51% of women, 10% men), it would appear that absolute strength rather than relative grip strength reduction may be more important for remaining independent in daily living in the elderly.

To identify people with a clinically meaningful reduction in handgrip strength in our sample of Swiss older adults, we applied cut-off values for detection of people at risk for mobility limitations, associated with sarcopenia/ dynapenia [2, 3]. According to cut off values published by Alley et al. [3] classifying people as weak (grip strength less than 26 kg for men and 16 kg for women), 22.7% male and 18% female participants in our sample were in this category. These individuals have a 7.6 (men) and 4.4 (women) times increased risk for mobility limitations, compared to older people with normal strength. In addition, 35.3% of the women and 21.6% of the men had “intermediate strength” (cut-off thresholds of 32 kg in men and 20 kg in women), with concomitant 3.6 (men) and 2.4 (women) times higher risk of impairment compared to older adults with normal strength values. In total, the percentage of participants with reduced strength according the proposed thresholds is 44% in men and 53% in women. Comparing the men and women with normal strength to the at risk of mobility limitation groups regarding dependency in daily living, those with normal strength were more than 2–5 times less likely living in a care home facility.

Even though cut-off values are not confirmed to be valid in detecting mobility limitations in the Swiss population yet, these results might give insight into current physical health and might indicate future need for help and care in the Swiss population.

In this study, handgrip strength was evaluated in two urban regions of German-speaking Switzerland with comparable handgrip strength observed at both sites. The age- and gender- distribution, as well as the percentage of people dependent in daily activities, were comparable with the Swiss population of the same geographic region [19, 20]. Hence, grip strength values reported in this study are likely representative of the urban, German-Swiss population. We cannot rule out, however, that handgrip strength may differ in French-, Italian- and Romansh-speaking areas of Switzerland.

### Limitations

This study recruited people from urban rather than rural areas of Switzerland. As people from rural backgrounds have been shown to have greater grip strength than those from urban environments [45], reference values in this study may be viewed as lower estimates of grip strength in the Swiss population. It is noteworthy, however, that our data are comparable with previously published grip strength in Swiss adults (aged 75–85) which included urban, suburban and rural populations.

Secondly, our study included people of various health status. Grip strength differences between men and women might therefore be different to results of other authors that included only healthy people [24, 46], since reduced wellbeing could influence the range of handgrip strength values and change the sex differences.

## Conclusion

This study reports the age- and sex-stratified reference values for handgrip strength in a representative sample of the Swiss population, aged 75–99 years. Grip strength decreased with age in both sexes with the relative decline being greater in men than in women. Nonetheless, men had significantly higher grip strength values in all age groups. While the Swiss population sample had a greater grip strength than that reported in other European countries, 44% (men) and 53% (women) were still classified as being at risk of developing mobility limitations.

## Abbreviations

ADL: Activities of daily living
CI: Confidence interval
IADL: Instrumental activities of daily living
MMSE: Mini Mental State Exam
SD: Standard deviation
SEM: Standard error of the mean
